# Investigation of the Neuroprotective Action of Japanese Sake Yeast on Dementia Type of Alzheimer Disease in Rats: Behavioral and Neurobiochemical Assessment

**DOI:** 10.3390/neurosci4010006

**Published:** 2023-01-19

**Authors:** Motahareh Haghipanah, Fatemeh Ghalami, Maryam Saadat, Saeid Abbasi-Maleki, Reza Hossein Gholizadeh Salmani, Thomas Budde, Nasrollah Moradikor

**Affiliations:** 1 Department of Neuroscience Research, Institute for Intelligent Research, 0105 Tbilisi, Georgia; 2 Ramsar International Branch, Mazandaran University of Medical Sciences, Sari 4691710001, Iran; 3 Department of Anatomical Sciences, Faculty of Medicine, Semnan University of Medical Sciences,Semnan 3514799442, Iran; 4 Pharmaceutical Sciences Research Center, Health Institute, Kermanshah University of Medical Sciences, Kermanshah 6715847141, Iran; 5 Department of Pathobiology, Faculty of Veterinary Medicine, Urmia Branch, Islamic Azad University, Urmia P.O. Box 969, Iran; 6 Institut für Physiologie I, Westfälische Wilhelms-Universität, 48149 Münster, Germany

**Keywords:** Alzheimer, anxiety, antioxidant, BDNF, inflammation

## Abstract

Dementia involves several factors, and it is required to administer an agent with several efficiencies for its treatment. Sake is known to have antioxidant and anti-inflammatory properties and improves the serum concentration of BDNF. This study aimed to evaluate the neuroprotective action of Japanese sake yeast on dementia of the Alzheimer disease type in rats by behavioral evaluation and neurobiochemical assessment. The rats were grouped as non-Alzheimer rats (control rats) and Alzheimer rats administrated with 0 (AD), 10 (10-AD), 20 (20-AD), 30 (30-AD), and 40 mg/kg (40-AD) of sake. Anxiety-like and depression-like behaviors, the concentrations of brain-derived neurotrophic factor (BDNF), malondialdehyde (MDA), and ferric reducing ability of plasma (FRAP) were evaluated. The expressions of IL-1β, TNF-α, and IL-6 were assessed. The results showed that Alzheimer disease caused anxiety-like and depression-like behaviors (*p* = 0.000), decreased the concentrations of BDNF (*p* = 0.000) and FRAP (*p* = 0.000), increased the concentration of MDA (*p* = 0.000), and increased the expressions of IL-1β (*p* = 0.000), TNF-α (*p* = 0.000), and IL-6 (*p* = 0.000). The results showed that oral gavage of sake in higher doses decreased anxiety-like and depression-like behaviors (*p* = 0.000), increased the concentrations of BDNF (*p* = 0.000) and FRAP (*p* = 0.000), and reduced the concentration of MDA (*p* = 0.000) and the expressions of IL-1β (*p* = 0.000), TNF-α (*p* = 0.000), and IL-6 (*p* = 0.000). In sum, Japanese sake yeast can have roles in treating dementia of the Alzheimer disease type, but its mechanisms must be assessed in future studies.

## 1. Introduction

Dementia is defined as a pathological, neurodegenerative process leading to decreased cognitive and functional abilities. It has several causes, diverse manifestations, heterogeneity depending on gendered risk factors, and diverse outcomes [1]. It has been reported that 40 million people over 65 years of age suffer from the disease, and 70% of them are influenced by Alzheimer’s disease [2]. The disease is known to cause early symptoms of insidious behavioral and personality changes and problems with language [3]. The symptoms of behavioral and personality changes in the disease are similar to those in psychiatric disorders [4]. A relation has been reported between anxiety and cognitive decline/dementia [5]. Studies have also reported significant changes in peripheral serum brain-derived neurotrophic factor (BDNF) at the late stage of the dementia spectrum [6]. Decreases in the concentrations of antioxidant factors in patients with dementia has been reported [7]. Several studies have reported increases in the concentrations of inflammatory and pro-inflammatory factors [8,9,10]. Therefore, dementia involves several factors, and it is required to administer an agent with several efficiencies for its treatment. Natural agents have been used for the treatment of diseases.

Sake yeast has been utilized for the production of alcoholic beverages since ancient times and is known for antioxidant properties [11]. Sake is produced via a brewing process, where starch is converted into sugars that ferment into alcohol. It was reported that the administration of sake decreases behavior signs, oxidative indices, and inflammatory responses in stressed rats [12]. Studies have also reported that consumption of sake can decrease intake and reduce formation of aberrant crypt foci by suppressing inflammation and oxidation-induced cell cycle disturbances [13].

Animal models are valuable tools for assessing new therapeutic strategies for the treatment of human diseases, as well as for studying the pathological mechanisms involved in disease processes. Initially, the rat was used as a testing species, but during the last decade, increasing knowledge of advanced genetic techniques developed in the mouse. 

In sum, dementia is closely related to anxiety and depression and the decrease in antioxidant factors and inflammation. On the other hand, sake improves antioxidant properties, decreases inflammation, and improves the serum concentration of BDNF. However, studies have not evaluated the effects of Japanese sake yeast on dementia of the Alzheimer disease type. This study evaluates the neuroprotective action of Japanese sake yeast on dementia of the Alzheimer disease type in rats by behavioral evaluation and neurobiochemical assessment.

## 2. Materials and Methods 

### 2.1. Materials

Sake yeast powder (GSP6) was prepared as a food supplement by Lion Corporation, Odawara-shi, Japan.

### 2.2. Animals

This study was conducted according to the recommendations given by the Ethics Committee of the International Center for Neuroscience Research. Ninety female rats with a weight of 220 ± 10 g were grouped into six groups, with 15 in each. The rats were grouped as non-Alzheimer rats lacking surgery (control rats) and Alzheimer rats administrated with 0 (AD), 10 (10-AD), 20 (20-AD), 30 (30-AD), or 40 mg/kg (40-AD) of sake. The doses were selected based on previous studies, and rats received them for two weeks [11]. The rats had free access to water and feed, and their rearing condition was constantly controlled. The sake was dissolved in water and administrated daily via oral gavage.

### 2.3. Surgery and Drug Administration

To induce anesthesia, the rats were intraperitoneally administrated with 90 mg/kg ketamine HCl and 10 mg/kg xylazine. The rats were then fixed in a stereotaxic apparatus (Narishige, Tokyo, Japan), and oligomers Aβ1-42 (1 μg/μL in each site) was infused into the hippocampal CA1 area bilaterally at a rate of 1 μL/5 min with the help of a 10 μL Hamilton syringe connected to an infusion pump, as reported by others [14]. Following infusion, the cannula was left in place for an additional 3 min to allow the complete diffusion of the drug. Infusion was performed with coordinates of AP = −4.8 mm from the bregma, ML = ±3.5 mm, and DV = −4 mm from dura mater. A recovery period (10 days) was considered for all the rats.

### 2.4. Behavioral Tests

#### 2.4.1. Open-Field Test (OFT)

The test was performed to evaluate anxiety based on other studies with the help of a dark area (72 × 72 × 45 cm) for 20 min [15].

#### 2.4.2. Elevated Plus Maze (EPM)

The test was utilized to evaluate anxiety with the help of apparatuses consisting of two open arms (50 cm × 10 cm) and two enclosed arms (50 cm × 10 cm, surrounded by 40 cm high wooden walls), raised 50 cm above the floor [15].

#### 2.4.3. Force Swimming Test (FST)

The test was conducted to evaluate depression with the help of a cylindrical swimming tank (50 cm high, 25 cm diameter) filled with 25 °C water for 15 min, as described by previous studies [16]. Immobility, swimming, and climbing were evaluated.

### 2.5. The Assessment of BDNF and Antioxidant-Associated Factors

The animals were decapitated, and the prefrontal cortex was isolated and frozen at −80 °C. Tissue was homogenized in cold lysis buffer, and BDNF was assessed by ELISA kits (Hangzhou Eastbiopharm Co., LTP, Hangzhou, China) following manufacturer instructions. Brain sections were frozen, homogenized, and investigated for malondialdehyde (MDA), and the ferric reducing ability of plasma (FRAP) was also assessed by ELISA kits (Hangzhou Eastbiopharm Co., LTP) as recommended by producer companies.

### 2.6. The Expression of Inflammatory Factors

Real-time PCR reactions were performed as reported by previous studies [17]. In summary, total ribonucleic acid (RNA) was extracted using a Qiagen Rneasy Mini Kit (Qiagen, Valencia, CA, USA). A reverse transcriptase (TaKaRa Biotechnology, Otsu, Japan) was used to synthesize complementary deoxyribonucleic acid (cDNA). Temperatures included initial denaturation at 95 °C for 30 s, 40 cycles of denaturation at 95 °C for 5 s, and annealing at 60 °C for 34 s. The expression levels were normalized compared with β-actin. Primer sequences were as follows: β-Actin: forward (TGGCACCCAGCACAATGAA), reverse (CTAAGTCATAGTCCGCCTAG); TNF-α: forward (CCCATGTTGTAGCAAACCCTC), reverse (TATCTCTCAGCTCCACGCCA); IL-1β: forward (CCACCTCCAGGGACAGGATA), reverse (TGGGATCTACACTCTCCAGC); and IL-6: forward (CAATGAGGAGACTTGCCTGG), reverse (TGGGTCAGGGGTG). All the primers were in directions 3–5.

### 2.7. Data Analysis

The data were investigated for normality, and because the data were normal, a parametric test of ANOVA was used. The Duncan test was used to compare between groups. A *p* < 0.05 was considered as significant. The data were analyzed using the Graph Pad Prism software (version 6.07).

## 3. Results

### 3.1. Anxiety-like Behaviors

**Figure 1:**
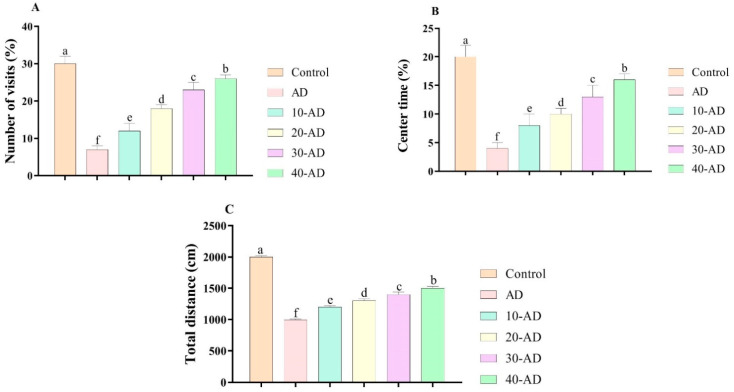
The effects of sake yeast on anxiety-like behaviors in rats with Alzheimer. (**A**) Number of visits; (**B**) Center time and (**C**) total distance. Different superscript symbols (a–f) show significant differences between groups at *p* < 0.05.

**Figure 2:**
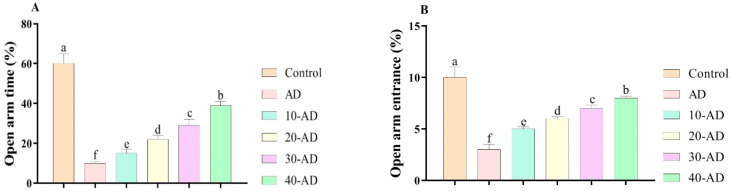
The effects of sake yeast on plus-maze behaviors in rats with Alzheimer. (**A**) Open arm time and (**B**) Open arm entrance. Superscript symbols (a–f) show significant differences between groups.

Figure 1 depicts the results for the effects of sake yeast on anxiety-like behaviors in rats with Alzheimer disease. The results show that Alzheimer disease reduces the number of visits (*p* = 0.000), center time (*p* = 0.000), and total distance (*p* = 0.000). This means that Alzheimer disease induces anxiety-like behaviors in rats. However, the results showed that oral administration of sake increased the number of visits (*p* = 0.000), center time (*p* = 0.000), and total distance (*p* = 0.000) in a dose-dependent manner. The results in Figure 2 agree with the obtained results in Figure 1. The results showed that the administration of sake increased open arm test results (*p* = 0.000) in a dose-dependent manner. In other words, oral administration of sake decreased anxiety-like behaviors.

### 3.2. Depression-like Behaviors

**Figure 3:**
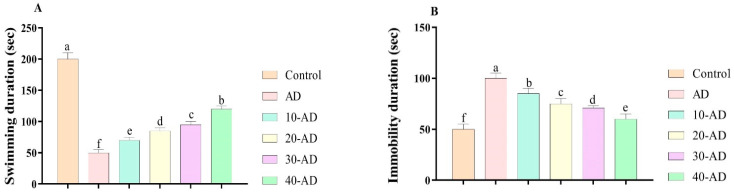
The effects of sake yeast on depression-like behavior in rats with Alzheimer. (**A**) Swimming duration and (**B**) Immobility duration. Superscript symbols (a–f) show significant differences between groups.

Figure 3 shows the effects of oral administration of sake on depression-like behavior. Alzheimer disease reduced swimming duration (*p* = 0.000) and increased immobility duration (*p* = 0.000). The administration of sake increased swimming duration (*p* = 0.000) and reduced immobility duration (*p* = 0.000). It must be mentioned that oral administration of sake reduces depression in rats with Alzheimer.

### 3.3. Biochemical Parameters

**Figure 4:**
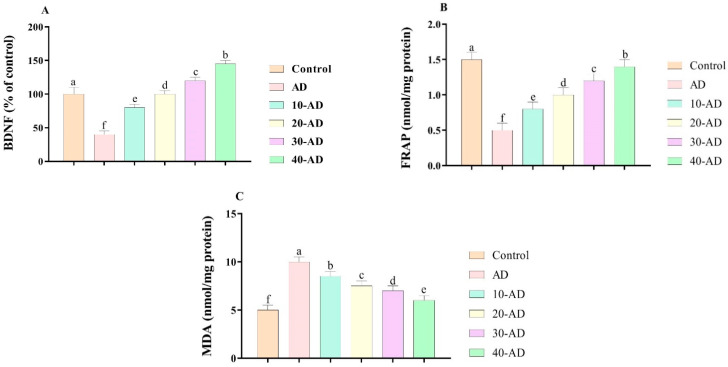
The effects of sake yeast on biochemical parameters in rats with Alzheimer. (**A**) BDNF; (**B**) FRAP and (**C**) MDA. Superscript symbols (a–f) show significant differences between groups.

Figure 4 shows the effects of sake yeast on biochemical parameters in rats with Alzheimer. The results show that Alzheimer disease reduces the concentrations of BDNF and FRAP while increasing the concentration of MDA. However, the results show that oral administration of sake increased the concentrations of BDNF (*p* = 0.000) and FRAP (*p* = 0.000) and decreased the concentration of MDA (*p* = 0.000) in a dose-dependent manner. 

### 3.4. The Expression of Inflammatory Factors

**Figure 5:**
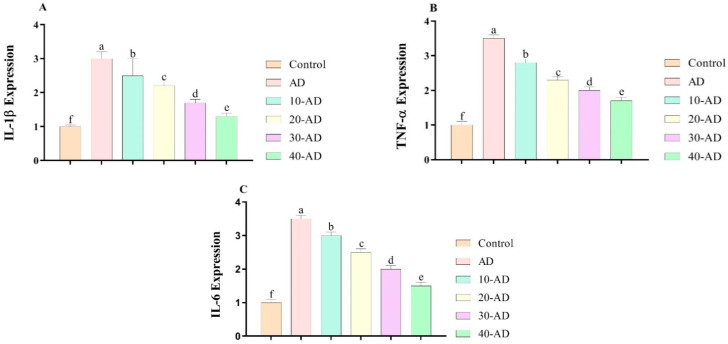
The effects of sake yeast on the expression of inflammatory factors in rats with Alzheimer. (**A**) IL-1β; (**B**) TNF-α and (**C**) IL-6. Superscript symbols (a–f) show significant differences between groups. Different superscript symbols (a–f) show significant differences between groups at *p* < 0.05.

Figure 5 depicts the effects of oral administration of sake on the expression of inflammatory factors. The results showed that Alzheimer disease increased the expression of inflammatory factors. This indicates a close relationship between Alzheimer disease and inflammatory factors. However, the results show that oral administration of sake decreased the expressions of IL-1β (*p* = 0.000), TNF-α (*p* = 0.000), and IL-6 (*p* = 0.000) in a dose-dependent manner.

## 4. Discussion

This study investigated the neuroprotective action of Japanese sake yeast on dementia of the Alzheimer’s disease type in rats via behavioral and neurobiochemical assessment in rats. The rat is one of the most commonly used experimental animal species in biomedical research, and because of its relevance to human physiology, the rat may provide highly predictable models for research in the pharmaceutical industry. The results showed that Alzheimer’s disease was closely related to anxiety and depression, which is in agreement with other studies [18,19]. It was reported that Alzheimer’s disease increases anxiety and depression by increasing amyloid-β [19]. In addition, inflammation and oxidation promote the progression of disease and anxiety and depression [20,21]. The results showed that the administration of sake reduced anxiety and depression. The mechanism of action of sake yeast in decreasing depression-like and anxiety-like behaviors can be associated with sake’s effects on BDNF and antioxidant factors, as will be discussed. The effects of antioxidant and anti-inflammatory factors in decreasing anxiety and depression have been previously reported [22,23].

The results showed that Alzheimer’s disease reduced the concentration of BDNF. The results are in agreement with previous studies on the relation between Alzheimer’s disease and the concentration of BDNF [24,25]. BDNF is an important member of the typical neurotrophin family of growth factors, nerve growth factor, and neurotrophins. It was reported that amyloid-β protein might prevent the proteolytic conversion of BDNF from pro-BDNF and reduce the concentration of BDNF [25]. In the current study, the amyloid-β protein was not assessed, and the relation between BDNF, amyloid-β protein, and Alzheimer’s disease must be cautiously discussed. The results showed that oral administration of sake increased the concentration of BDNF. The results agree with previous studies on the effects of sake on the expression of BDNF [11]. The mechanism of sake in increasing BDNF concentration is still not clear. However, decreased inflammation and increased antioxidant status could be considered mechanisms for the effects of sake in increasing BDNF concentration. It was reported that an increase in antioxidants [26,27] and a decrease in inflammation [28,29] are closely related to BDNF.

The results showed that Alzheimer’s disease reduced MDA and increased FRAP. MDA is an end product of lipid peroxidation and a side product of thromboxane A2 synthesis [30]. FRAP is utilized to determine antioxidant activity. The results show that Alzheimer’s causes oxidation and reduces antioxidant capacity. Several studies have reported poor antioxidant status in patients with Alzheimer’s disease [31,32]. Oxidative damage due to reactive oxygen species has been shown in the pathogenesis of neurodegenerative diseases [33]. The results showed that the administration of sake improved antioxidant status in a dose-dependent manner. Studies have reported a reverse relation between MDA and FRAP [34]. The results for the effects of sake on antioxidant status agree with previous studies [11]. They reported that sake yeast shows antioxidant activity via antioxidant enzyme activities and decreases oxidative stress. The antioxidant activity of sake can be attributed to its compounds because it works dose-dependently.

The results show that Alzheimer’s increases the expression of inflammatory factors. The results are in agreement with other studies indicating a closed relation between inflammation and Alzheimer’s disease [35,36]. The disease works via inflammatory pathways. IL-6, IL-1β, and TNF-α increase inflammation and closely relate to other inflammatory factors [37,38,39,40]. However, the administration of sake in a dose-dependent manner reduced the inflammation. The results are in agreement with previous studies regarding the anti-inflammatory effects of sake [13]. Sake working as an anti-inflammatory compound could be attributed to its structure.

## 5. Conclusions

This study evaluated the effects of Japanese sake yeast on dementia of the Alzheimer’s disease type in rats. It showed that Japanese sake yeast reduces anxiety-like and depression-like behaviors and works as an antioxidant and anti-inflammatory agent. In sum, Japanese sake yeast can have roles in treating dementia of the Alzheimer’s disease type but must be evaluated by other mechanisms.

## Data Availability

The datasets used and analyzed during this study are available from the corresponding author on reasonable request.
